# Determinants of Serum Concentrations of Lipopolysaccharide-Binding Protein (LBP) in the Adult Population: The Role of Obesity

**DOI:** 10.1371/journal.pone.0054600

**Published:** 2013-01-22

**Authors:** Arturo Gonzalez-Quintela, Manuela Alonso, Joaquin Campos, Luis Vizcaino, Lourdes Loidi, Francisco Gude

**Affiliations:** 1 Department of Internal Medicine, Complejo Hospitalario Universitario, Santiago de Compostela, Spain; 2 Department of Biochemistry, Complejo Hospitalario Universitario, Santiago de Compostela, Spain; 3 Fundación Pública Galega de Medicina Xenómica, Santiago de Compostela, Spain; 4 Department of Clinical Epidemiology, Complejo Hospitalario Universitario, Santiago de Compostela, Spain; Centers for Disease Control and Prevention, United States of America

## Abstract

**Background and Aim:**

Assessment of serum concentration of lipopolysaccharide (LPS)-binding protein (LBP) has been suggested as a useful biomarker to indicate activation of innate immune responses to microbial products. We investigated LBP concentrations and associations with demographics, lifestyle factors, and common metabolic abnormalities in adults. We also examined if LBP concentrations were associated with common polymorphisms in genes coding for LBP (rs2232618), CD14 (rs2569190), and TLR4 (rs4986790), the molecules responsible for the innate immune response to LPS, or serum levels of soluble CD14 (sCD14) and proinflammatory cytokines.

**Methods:**

Serum LBP was measured with a commercial immunoassay in a random sample of the adult population (n = 420, 45% males, age 18–92 years) from a single municipality.

**Results:**

Serum LBP concentrations increased with age (P<0.001) and were higher in individuals who were overweight or obese than in normal-weight individuals (P<0.001). Similarly, LBP concentrations were higher in individuals with metabolic syndrome than in individuals without it (P<0.001). Among metabolic syndrome components, LBP concentrations were independently associated with abdominal obesity (P = 0.002) and low concentrations of HDL-cholesterol (P<0.001). Serum LBP concentrations tended to be independently associated with smoking (P = 0.05), but not with alcohol consumption. Likewise, there was not significant association between LBP concentrations and gene polymorphisms. Concentrations of LBP significantly correlated with serum levels of proinflammatory cytokines (IL-6 and IL-8), sCD14, and with liver enzymes.

**Conclusions:**

Serum LBP concentrations increased with age. Overweight, obesity, and having metabolic syndrome (particularly, low HDL cholesterol levels) were associated with higher LBP concentrations. These findings are consistent with microbial exposure playing a role in these inflammatory, metabolic abnormalities.

## Introduction

A dysregulated response to bacteria or their products such as lipopolysaccharide (LPS, endotoxin) underlies many common inflammatory diseases [1], [2]. Like all mammals, humans are equipped with an LPS-sensing machinery consisting primarily of LPS-binding protein (LBP); CD14, a glycosylphosphatidyl-inositol (GPI)-anchored monocyte differentiation antigen; and toll-like receptor 4 (TLR4), a signal-transducing integral membrane protein [2]. CD14 also exists in a soluble form (sCD14), a 43–53 kD glycoprotein that derives from either protease-mediated membrane CD14 shedding [3] or liver synthesis as a type II acute-phase reactant [4]. LBP is a 58-kDa glycoprotein synthesized in the liver that is released into circulation as a type I acute-phase reactant [5]–[7]. Levels of LBP peak in serum shortly after bacteremia or endotoxemia, and remain increased up to 72 hours later [8], [9]. Once in the circulation, LBP forms a complex with LPS that enhances the binding of LPS with CD14 receptors [6]–[8]. Membrane CD14 is associated with TLR4, which transduces a signal from the CD14-bound LPS to the cell nucleus, triggering a cascade of inflammatory cytokines [10]. Some of these cytokines, specifically IL-1 and IL-6, induce the synthesis of acute-phase proteins in the liver [11]. Membrane CD14, sCD14, and LBP thus participate in a complex mechanism of immune regulation involving both up-regulation and down-regulation of the inflammatory process triggered by LPS. Some studies have demonstrated a concentration-dependent dual role of LBP in the pathogenesis of Gram-negative sepsis: low concentrations of LBP enhance the LPS-induced activation of mononuclear cells, whereas the acute-phase rise in LBP concentrations inhibits LPS-induced cellular stimulation [12]. It should be also noted that LBP, a soluble pattern recognition molecule, appears to be able to bind bacterial compounds other than LPS [8], [13].

Assessment of LBP concentrations in serum or plasma has been suggested as a useful marker during systemic infectious complications [8], [14]. Compared with other acute phase reactants, the rise of LBP is relatively slow [8], and thus can serve to monitor the interaction between bacterial compounds (particularly, LPS) and innate immunity cells taking place over time [15]. The measurement of bacterial products such as LPS in biologic fluids has significant limitations [16], [17]; therefore, the LBP level has been suggested as a clinical marker of “effective endotoxemia” [18]–[22]. Studies of the distribution of LBP in the general population are scarce. These studies may serve to identify how serum markers are associated with demographic variables (such as age, sex or ethnicity), lifestyle factors (such as alcohol consumption and smoking), and common metabolic abnormalities (such as obesity and components of metabolic syndrome). These associations may be relevant for interpreting LBP concentrations in clinical settings. Serum levels of LBP are increased in heavy drinkers, probably reflecting high LPS exposure due to alcohol-induced damage of the gastrointestinal barrier [23], [24]. In previous studies we observed that heavy drinkers with a common single nucleotide polymorphism (SNP) in the CD14 promoter gene had higher levels of serum LBP [25]. The potential influence of smoking on LBP concentrations has been not investigated. Serum LBP levels were increased in patients with morbid obesity undergoing surgery, particularly in those with steatohepatitis [20]. Likewise, serum LBP concentrations were associated with obesity and related metabolic disorders in a selected sample of apparently healthy Chinese subjects [18].

The present study aimed to investigate LBP concentrations in a general adult population and the potential relationships with demographic factors, lifestyle factors, and common metabolic abnormalities. In addition, we investigated whether LBP levels were associated with common SNPs in molecules at the interface of the innate immune response to LPS (CD14, TLR4, and LBP itself), serum concentrations of sCD14, and proinflammatory cytokine levels.

## Methods

### Study Population and Design

This cross-sectional study was included in a survey of the general population in the municipality of A-Estrada (Spain), as detailed elsewhere [26]. Briefly, an age-stratified random sample (n = 720) of the adult (>18 years) population of the municipality was drawn from the Health Care Registry, which included >95% of the population. A total of 469 individuals consented to participate in the study. A frozen serum sample (obtained under fasting conditions) for LBP determination was available for 420 individuals (all Caucasians, median age 55 years, range 18–92 years, 45.0% males).

### Ethics Statement

The source study (FIS1306/99) was reviewed and approved by the Institutional Review Board of the Complejo Hospitalario Universitario from Santiago de Compostela (Spain). The present study (PGIDIT06PXIB918313) was reviewed and approved by the Clinical Research Ethics Committee from Galicia (Spain). Written informed consent was obtained from each participant in the study, which conformed to the current Helsinki Declaration.

### Lifestyle Factors

All individuals underwent a physician-administered questionnaire. Alcohol consumption was evaluated as the number of standard drinking units (glasses of wine [∼10 g], bottles of beer [∼10 g], and spirits [∼10 g]) regularly consumed per week. Individuals were classified as abstainers/occasional drinkers (<10 g/week), light-moderate drinkers (10–200 g/week) or heavy drinkers (≥210 g/week). Consumers of at least one cigarette per day were considered smokers.

### Metabolic Abnormalities

Body mass index (BMI) was calculated as the weight (in kilograms) divided by the square of height (in meters). Individuals were classified according to BMI as normal-weight (<25 kg/m^2^), overweight (25–30 kg/m^2^), or obese (>30 kg/m^2^). According to the Adult Treatment Panel III [27], individuals were considered to have metabolic syndrome when at least three of the following criteria were present: abdominal obesity (waist circumference >102 cm in males or >88 cm in females); hypertriglyceridemia (serum triglycerides ≥150 mg/dL); low levels of high-density lipoprotein-cholesterol (HDL, <40 mg/dL in males or <50 mg/dL in females); high blood pressure (≥130/≥85 mmHg or current anti-hypertensive medication use); and hyperglycemia (blood glucose ≥110 mg/dL or current use of anti-diabetic therapy).

### Determination of Serum Levels of Lipopolysaccharide-Binding Protein (LBP)

Serum LBP was measured by chemiluminescent enzyme immunoassay (Immulite, Siemens Medical Solutions, Gwynedd, UK). The upper limit of the calibration range is 200 µg/mL and the analytical sensitivity is 0.2 µg/mL. The intra- and interassay coefficients of variation for the assay were <11%. According to the manufacturer, a study performed on 160 apparently healthy volunteers yielded a mean of 5.3 µg/mL, a 95^th^ percentile of 8.4 µg/mL and an absolute range of 2.0 to 15.2 µg/mL.

### Additional Laboratory Determinations

Serum levels of liver enzymes were measured in an Olympus AU-400 analyzer (Olympus, Tokyo, Japan). The serum levels of gamma-glutamyl transferase (GGT) in this population have been reported elsewhere [28]. Serum levels of proinflammatory cytokines (IL-6, IL-8, and TNF-alpha) were measured by a chemiluminescent enzyme immunoassay (Immulite) and have been reported elsewhere [29]–[31]. Serum concentrations of soluble CD14 (sCD14) were measured by means of a commercial enzymoimmunoassay (Quantikine, R&D Systems, Minneapolis, USA).

### Single Nucleotide Gene Polymorphisms (SNPs)

DNA was extracted from peripheral blood leukocytes using standard protocols. The SNPs in the LBP, CD14 and TLR4 genes were genotyped using TaqMan™ validated assays (Applied Biosystems, Foster City, CA). The polymorphisms studied included LBP +1306T/C (rs2232618, p.Phe436Leu), CD14 -159C/T (rs2569190), and TLR4+896A/G (rs4986790, p.Asp299Gly). The LBP rs2232618 is a functional SNP that has been associated with host susceptibility to sepsis and multiple organ dysfunction in patients with major trauma [32]. The rs2569190 SNP in the CD14 promoter has been associated with a gain of function [33]. Its frequency in this population has been reported elsewhere [34]. The TLR4 rs4986790 SNP has been associated with hyporesponsiveness to LPS [35].

### Statistical Analyses

The Mann-Whitney U-test and the Jonckheere-Terpstra trend test were used to compare LBP concentrations among groups. The Spearman’s rank test was used to assess correlations. Linear regression was used for multivariate analyses. For that purpose, LBP concentrations (dependent variable) were log_10_-transformed to normalize the distribution. P-values lower than 0.05 were considered statistically significant.

## Results

**Figure 1:**
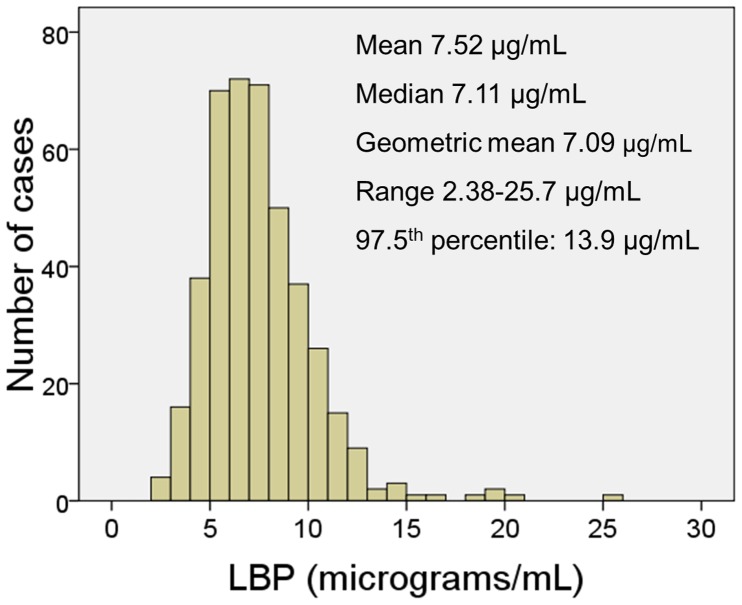
Histogram of serum concentrations of lipopolysaccharide-binding protein (LBP) in the study population.

**Figure 2:**
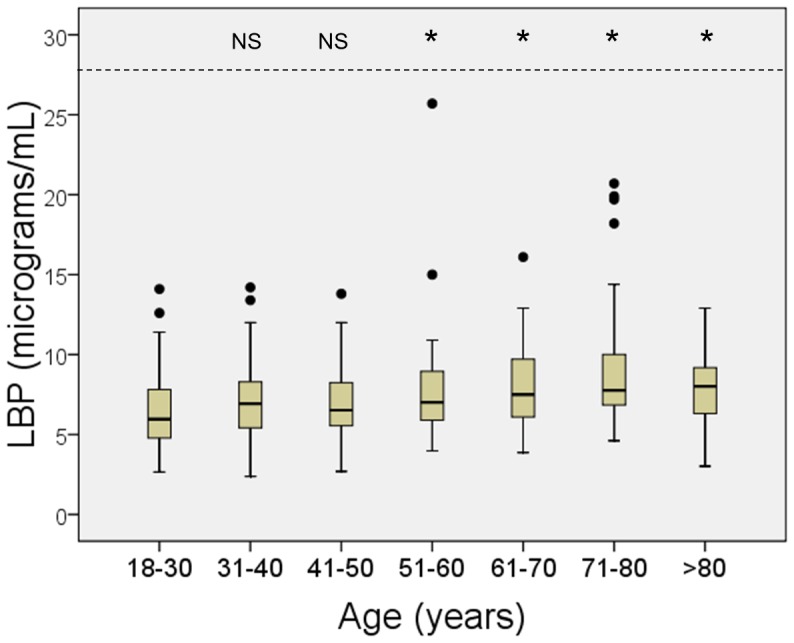
Serum concentrations of lipopolysaccharide-binding protein (LBP) in the different age strata. Horizontal lines represent median values, boxes represent the interquartile range, whiskers represent the range, and dots represent extreme values (higher than the 75^th^ percentile plus 1.5 times the interquartile range). The P-values (top of the figure) reflect comparisons to the group of 18–30 year-old (Mann-Whitney test). NS, not significant (P>0.05); *, P<0.005.

The distribution of serum LBP concentrations in this population is shown in Figure 1. Serum LBP concentrations steadily increased with age from a median of 5.96 µg/mL in individuals 18-to-30 years old to a median of 8.29 µg/mL in those >80 years old (P for trend, <0.0001, Figure 2). The increase in serum LBP was statistically significant (P<0.05) in individuals above 50 years old (Figure 2); therefore, the age of 50 years was used for stratification in some analyses.

**Figure 3:**
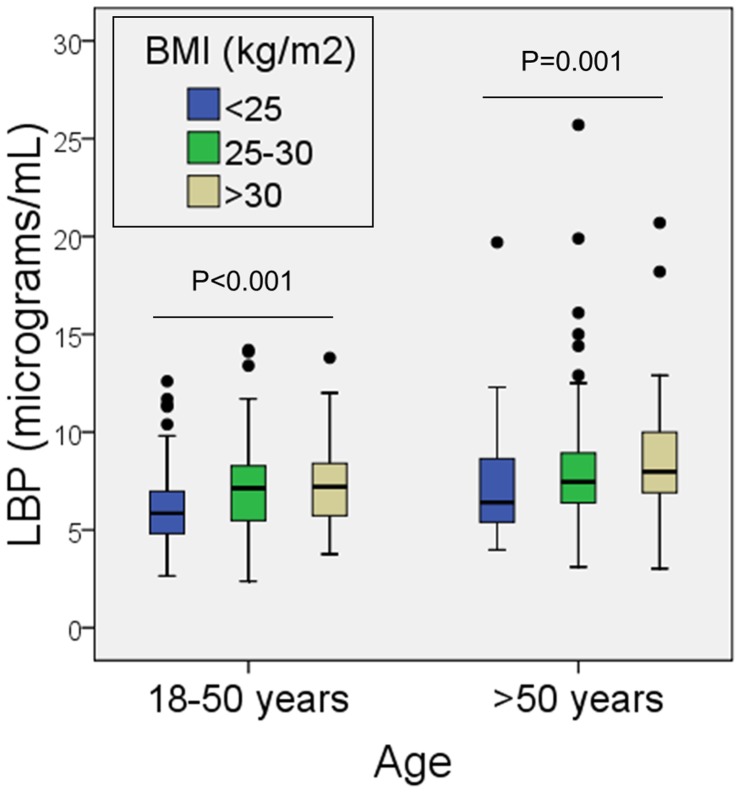
Serum concentrations of lipopolysaccharide-binding protein (LBP) according to age strata and body mass index (BMI). Horizontal lines represent median values, boxes represent the interquartile range, whiskers represent the range, and dots represent extreme values (higher than the 75^th^ percentile plus 1.5 times the interquartile range). P-values we obtained with the Jonkheere-Terpstra test for trend.

**Figure 4:**
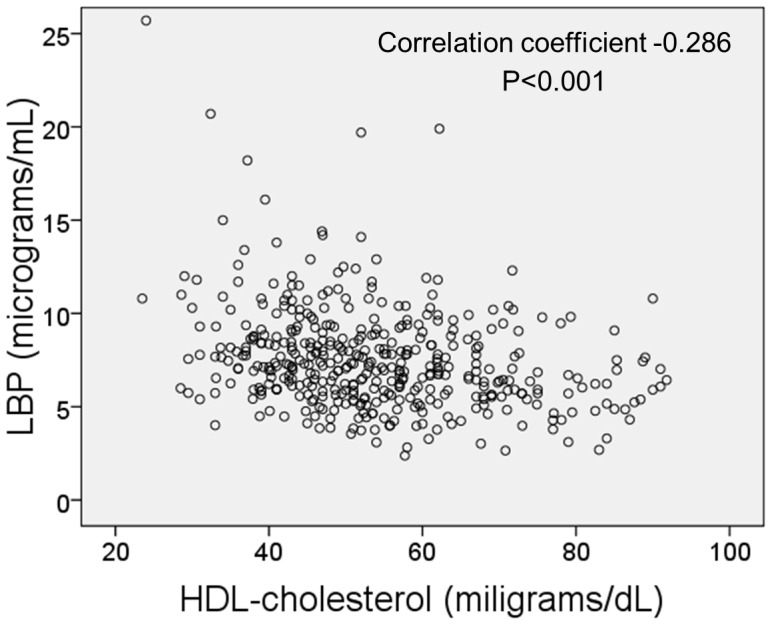
Scatterplot of individual concentrations of lipopolysaccharide-binding protein (LBP) and HDL-cholesterol. Correlation coefficient was obtained with the Spearman’s rank test.

In univariate analyses, overweight and obese individuals had higher LBP concentrations than normal-weight individuals. Similarly, individuals with metabolic syndrome had higher LBP concentrations than those without metabolic syndrome (Table 1). LBP was still associated with being overweight and obese after stratifying for age (Figure 3). Furthermore, there was a significant trend towards increased LBP concentrations with increasing categories of BMI (Figure 3).The linear relationship between body mass index and LBP was still present after adjusting for additional confounders such as gender, alcohol consumption and smoking (Table 2). Similarly, the association between metabolic syndrome and LBP concentration was independent of confounders (data not shown). Among the components of metabolic syndrome, serum LBP concentrations were independently associated with abdominal obesity and low HDL-cholesterol levels (Table 3). In fact, there was a significant negative correlation between serum LBP levels and serum HDL-cholesterol levels (Figure 4).

**Table 1:** Serum concentrations of lipopolysaccharide-binding protein (LBP) and soluble CD14 (sCD14) in relation to demographic variables, lifestyle factors, and metabolic abnormalities.

	No.	LBP (µg/mL)	sCD14 (µg/mL)
**Sex**			
Female (reference)	231	6.97 (5.71–8.55)	3.38 (2.89–3.80)
Male	189	7.39 (5.73–9.15)	3.30 (2.95–3.80)*
**Age**			
18–50 years (reference)	191	6.46 (5.24–8.24)	3.17 (2.77–3.62)
>50 years	229	7.58 (6.29–9.37)**	3.49 (3.08–3.94)**
**Alcohol consumption**			
Abstainers (reference)	195	7.07 (5.66–8.78)	3.23 (2.83–3.81)
Light-moderate drinkers	141	7.29 (5.54–8.82)	3.43 (2.94–3.80)
Heavy drinkers	84	7.06 (6.02–8.86)	3.33 (3.03–3.77)
**Smoking**			
Non-smokers (reference)	329	7.11 (5.85–8.74)	3.37 (2.90–3.80)
Smokers	91	7.18 (5.42–9.15)	3.23 (2.98–3.70)
**Body mass index**			
Normal weight (reference)	112	5.90 (5.09–7.67)	3.34 (2.89–3.80)
Overweight	180	7.29 (5.96–8.78)**	3.35 (2.91–3.84)
Obese	127	7.75 (6.35–9.47)**	3.34 (2.89–3.80)
**Metabolic syndrome**			
Absent (reference)	315	6.82 (5.48–8.40)	3.28 (2.89–3.76)
Present	105	8.02 (6.63–9.82)**	3.48 (3.09–3.89)

Data are medians and interquartile ranges (within parenthesis).

* P<0.05;

** P<0.001 (comparison with the reference category, Mann-Whitney test).

Body mass index was unavailable for one individual.

**Table 2:** Multivariate analysis of factors associated with serum lipopolysaccharide-binding protein (LBP) concentrations: demographic factors, lifestyle factors and body mass.

Covariates	Coefficient (slope)	Standard error	P-value
**Age** (years)	0.002	0.0003	<0.001
**Sex**			
Female	(reference)		
Male	0.024	0.016	0.12
**Alcohol consumption**			
Abstainers	(reference)		
Light-moderate drinkers	−0.022	0.016	0.16
Heavy drinkers	−0.017	0.021	0.40
**Smoking**			
Non-smokers	(reference)		
Smokers	0.036	0.018	0.05
**Body mass index** (kg/m^2^)	0.006	0.001	<0.001
**Constant (intercept)**	0.552	0.043	

Linear regression analysis. LBP concentrations (dependent variable) was log_10_-transformed in order to normalize their distribution. All listed covariates entered the equation. Age was introduced in years and BMI was introduced in kg/m^2^; the remaining variables were introduced as “1 = present or yes” and “0 = absent or not”. Complete data were available for 419 individuals. The model explained 13.6% of the variability of serum LBP concentrations (R square, 0.136).

**Table 3:** Multivariate analysis of factors associated with serum lipopolysaccharide-binding protein (LBP) concentrations: metabolic syndrome components.

Covariates	Coefficient (slope)	Standard error	P-value
**Abdominal obesity**			
Absent	(reference)		
Present	0.051	0.016	0.002
**Hyperglycemia**			
Absent	(reference)		
Present	0.005	0.017	0.78
**Low HDL-cholesterol**			
Absent	(reference)		
Present	0.083	0.016	<0.001
**Hypertriglyceridemia**			
Absent	(reference)		
Present	−0.008	0.018	0.65
**Hypertension**			
Absent	(reference)		
Present	0.003	0.019	0.87
**Constant (intercept)**	0.694	0.021	

Linear regression. LBP concentrations (dependent variable) was log_10_-transformed in order to normalize their distribution. Coefficients are adjusted for all listed variables, as well as for age and sex. Age was introduced in years; the remaining variables were introduced as “1 = present or yes” and “0 = absent or not”. Metabolic syndrome components were defined by the Adult Treatment Panel III criteria [27]: abdominal obesity (waist circumference >102 cm in males or >88 cm in females); hypertriglyceridemia (serum triglycerides ≥150 mg/dL); low levels of HDL-cholesterol (<40 mg/dL in males or <50 mg/dL in females); hypertension (≥130/≥85 mmHg or current use of anti-hypertensive medication use); and hyperglycemia (blood glucose ≥110 mg/dl or current use of anti-diabetic therapy). Data were available for all 420 individuals. The model explained 17.7% of the variability of serum LBP concentrations (R square, 0.177).

Male sex tended to be independently associated with higher serum LBP concentrations (Table 2). Alcohol consumption was not associated with LBP concentrations (Table 2). Smoking tended to be positively associated with LBP concentrations, particularly after adjusting for confounders (Table 2).

The LBP +1306T/C SNP (rs2232618) allele frequencies were 0.91 and 0.09, respectively. Carriers of the C allele tended to have higher serum LBP concentrations, although the difference was not statistically significant (P = 0.1, Table 4). Likewise, serum LBP concentrations were independent of the studied CD14 and TLR4 gene SNPs (Table 4). In contrast, serum sCD14 concentrations were higher among carriers of the T allele at the CD14-159 (rs2569190) SNP (Table 4).

**Table 4:** Serum concentrations of lipopolysaccharide-binding protein (LBP) and soluble CD14 (sCD14) in relation to common single nucleotide polymorphisms (SNPs) in the LBP, CD14, and TLR4 genes.

	No.	LBP (µg/mL)	sCD14 (µg/mL)
**LBP rs2232618 SNP**			
TT (reference)	196	7.03 (5.50–8.80)	3.27 (2.79–3.69)
CT or CC	41	7.39 (5.98–9.99)	3.34 (2.90–3.97)
**CD14 rs2569190 SNP**			
CC (reference)	63	7.34 (5.64–9.09)	3.19 (2.72–3.55)
CT or TT	190	7.03 (5.41–8.81)	3.38 (2.90–3.79)*
**TLR4 rs4986790 SNP**			
AA (reference)	232	7.09 (5.56–8.84)	3.30 (2.89–3.74)
AG or GG	21	7.14 (5.17–10.7)	3.15 (2.75–3.65)

Data are medians and interquartile ranges (within parenthesis).

DNA samples were available for 237–253 out of 420 individuals.

* P<0.05 (comparison with the reference category, Mann-Whitney test).

Serum sCD14 concentrations were found to be significantly correlated with those of LBP (correlation coefficient 0.149, P = 0.002). Similar to LBP, sCD14 concentrations increased with age. Contrary to LBP, sCD14 concentrations were not significantly associated either body mass index or metabolic syndrome (Table 1). Likewise, serum sCD14 concentrations were not significantly correlated with HDL-cholesterol (data not shown).

There was a statistically significant correlation between serum LBP concentrations and concentrations of proinflammatory cytokines, particularly IL-6 and IL-8 (Table 5). Similarly, there was a correlation between LBP concentrations and serum levels of liver enzymes, particularly gammaglutamyl transferase (GGT, Table 5). The correlation between LBP and GGT was stronger among obese individuals (correlation coefficient 0.215, P = 0.01) than compared with overweight (correlation coefficient 0.103, P = 0.16) and normal weight (correlation coefficient 0.025, P = 0.78) subjects.

**Table 5:** Correlation between serum lipopolysaccharide-binding protein (LBP) concentrations and concentrations of proinflammatory cytokines and liver enzymes.

	IL-6	IL-8	TNF-α	AST	ALT	GGT
**Correlation coefficient with LBP**	0.125	0.107	0.038	0.007	0.126	0.213
**P-value**	0.01	0.02	0.43	0.88	0.01	<0.001

IL-6, interleukin-6; IL-8, interleukin-8; TNF-α, tumor necrosis factor alpha; AST, aspartate aminotransferase; ALT, alanine aminotransferase; GGT, gamma-glutamyl transferase.

## Discussion

Lipopolysaccharide-binding protein (LBP) might be a biomarker to indicate activation of innate immune responses in response to microbial compounds, particularly LPS [8], [9], [14], [15]. The present study demonstrated that serum LBP concentrations (a) increased with age, (b) were higher in individuals who were overweight and obese compared with normal-weight individuals, (c) were higher in individuals with metabolic syndrome than in individuals without it, (d) were independently associated with the metabolic syndrome components, abdominal obesity and low concentrations of HDL-cholesterol, (e) tended to be independently associated with smoking, but not with alcohol consumption; (f) correlated with serum levels of proinflammatory cytokines (IL-6 and IL-8), and sCD14; and (g) were not significantly associated with specific LBP, CD14 or TLR4 SNPs.

The finding that LBP levels increased with age in adults has not been reported previously. On the contrary, a decrease of LBP with age was reported in a selected sample of individuals being studied for cardiovascular risk [36]. In our study in a general population, the LBP increase was steady through decades of age, and was statistically significant from the 6^th^ decade onwards with respect to the reference category of young adults. The mechanisms underlying such an increase are not known. Similar increases with age have been observed for other inflammatory markers in the same population [37]. The composition of intestinal microbiota, gut permeability, and subsequent bacterial translocation are critical determinants of LPS exposure in health and disease [38]–[40]. Intestinal permeability increases with age in experimental models [41]; although, this was not confirmed in human studies [42].

An increased BMI was strongly associated with increased serum levels of LBP in the general adult population studied. Alterations in gut microbiota and gut permeability to LPS are characteristics of obesity and related metabolic disorders [38], [43]–[45]. Obese mice develop intestinal bacterial overgrowth and display enhanced intestinal permeability [46]. Chronic low-grade inflammation induced by subclinical endotoxemia may be involved in the pathogenesis of metabolic disorders [43]–[45], [47]. Furthermore, lowering the LPS concentrations with antibiotics [44], [45] could improve metabolic outcomes. In healthy men, there is a link between food intake and plasma LPS [48]. Increased plasma LPS and LBP can be induced with a high-fat, high-carbohydrate meal [49]. Elevated LBP levels have been observed in Spanish patients with morbid obesity [20] and in Chinese volunteers from an obesity case-control study with restrictive selection criteria [18]. In addition, a positive correlation between LBP and BMI was reported in Spanish men with glucose intolerance [36]. The inflammatory consequences of enhanced LPS exposure in obese patients include non-alcoholic steatohepatitis [20], [50]. In line with this, serum LBP concentrations also correlated with liver enzyme levels, particularly with serum GGT, a marker of liver damage and indicator of liver involvement in systemic inflammatory disease [51]. Moreover, the correlation between LBP and GGT was highest among obese subjects. These findings are consistent with bacterial compounds playing a role in the development of non-alcoholic fatty liver disease in obese individuals [20], [50].

The association of LBP concentrations with low HDL-cholesterol, found in the present study, was independent of BMI and additional confounders. This finding confirms previous observations in selected samples [18], [52] and is consistent with HDLs playing a role in regulating the inflammatory response [2], [53]–[56]. HDLs have been shown to bind and neutralize LPS [53], [54]. Among plasma lipoproteins, HDL has the highest binding capacity for LPS [57]. In addition, LPS is transferred from HDLs to LDLs by LBP and additional transfer proteins [58]. In vitro, HDL-cholesterol can attenuate LPS-induced production of proinflammatory cytokines [59]. Thus, circulating levels of HDLs are reduced in sepsis/septic shock [55] and a low serum level of HDL-cholesterol is a poor prognostic factor for severe sepsis [59]. Low levels of HDL-cholesterol are a well-known, traditional risk factor for cardiovascular disease [56]. Enhanced LPS exposure has been recognized as a factor in cardiovascular disease [2], [22]. Future studies are needed to elucidate if low HDL-cholesterol concentrations may be one of the pathogenetic links between innate immune system activation and atherosclerosis.

Alcohol consumption was not associated with LBP levels in this study. Previous studies have shown that alcohol intake enhances intestinal permeability [24] and subsequent LPS absorption, as revealed by increased serum LPB levels [23]. Serum LBP levels reported for alcoholics [25] were higher than found for adults from the general population in the present study (median 21.4 µg/mL vs median 7.11 µg/mL, respectively). Possibly, the level of alcohol intake in this general population was not enough to demonstrate a significant influence on LBP concentrations. To the best of our knowledge, the association between smoking and increased LBP concentrations has not been described previously. Endotoxin (LPS) is present in tobacco smoke [60] and may be one of the factors that can promote inflammation in smokers, as reviewed elsewhere [61]. Previous studies have shown that LBP and sCD14 levels are increased in the bronchoalveolar lavage fluid of smokers [62]. This is in agreement with our findings, which should be confirmed in further studies because the association between smoking and LBP was of borderline significance and only evident after adjusting for confounders.

Serum LBP concentrations were associated with serum levels of proinflammatory cytokines (IL-6 and IL-8). This finding is consistent with the role of LBP as an acute phase response protein [5]–[8], [15]. Furthermore, serum LBP concentrations were correlated with serum sCD14, which can be also a reactant to metabolic endotoxemia [63]. However, serum sCD14 concentrations were associated with neither obesity nor related abnormalities. Although functionally linked, LBP and sCD14 are different type-I and type II, respectively, acute phase response proteins [4]–[7]. Taken together, these could indicate that obesity and related metabolic abnormalities are associated to specific innate inflammatory responses.

The human LBP gene is located on chromosome 20 [64], [65]. Intriguingly, the LBP gene shares an intron-exon pattern with other lipid-binding and transferring proteins on the same chromosome [64], [65]. Genetic variations in LBP may account for susceptibility and course of infectious diseases [8]. It has been recently reported that a functional SNP (rs2232618) in the LBP gene is associated with susceptibility to sepsis and multiple organ dysfunction in patients with major trauma [32]. However, we could not find a significant association between that SNP and LBP concentrations in the general population. Similarly, we found no association between LBP concentrations and common functional SNPs in the CD14 and TLR4 genes that can modify the host immune response to LPS [33], [35], and the risk for inflammatory conditions associated with LPS exposure [41], [66]–[68]. In a previous study we observed that both LBP and sCD14 concentrations were increased in alcoholics carrying the T allele at the CD14 -159 SNP [25]. In the present study, such genotype was associated with higher sCD14 levels but not with LBP levels.

Strength of this study is its population-based design including randomly selected individuals with a broad age range (18 to 92 years). Admittedly, the study has also limitations. First, the epidemiological, cross-sectional nature of this study does not allow for a causal inference and does not provide a mechanistic insight into the findings. Second, LBP concentrations were measured in samples that had been stored frozen for years. To the best of our knowledge, there is no available information about stability or instability of LBP in frozen samples over time. It should be noted that the observed measurements were similar to those reported in different populations using fresh samples, including those reported by the manufacturer in apparently healthy volunteers. Moreover, it should be noted that the study was not aimed to investigate absolute LBP concentrations but the association of LBP concentrations with demographic, lifestyle, and metabolic variables. Along this line, the potential misclassification bias would be non-differential, i.e., the unreliability in LBP measurements must be expected to be equal among obese and non-obese individuals. Therefore, it can only result in bias toward the null (type 2 error) and not a type 1 error, i.e. it could have obscured an effect but it could not cause a false effect to be seen. Third, blood levels of LPS were not measured in the present study because samples were neither obtained nor stored under nonpyrogenic, sterile conditions. It should be noted that LPS is not the only microbial ligand for LPS [8], [13], and that LBP increase is delayed with respect to LPS exposure [8], [9]. Hence, there was no correlation between blood LPS and LBP concentrations in previous studies [14].

The serum/plasma LBP commercial assay is intended as a tool for management of patients with infectious complications, because increased LBP concentrations may be indicative of exposure to bacterial compounds (particularly, LPS) and may be prognostic of disease progression [8], [14], [15], [18]–[23]. This study in an unselected adult population suggests that age and common conditions such as obesity and related metabolic disorders should be taken into consideration when interpreting LBP levels in clinical settings.
